# Spontaneous Regeneration of Plantlets Derived from Hairy Root Cultures of *Lopezia racemosa* and the Cytotoxic Activity of Their Organic Extracts

**DOI:** 10.3390/plants11020150

**Published:** 2022-01-06

**Authors:** Norely Vargas-Morales, Norma Elizabeth Moreno-Anzúrez, Janeth Téllez-Román, Irene Perea-Arango, Susana Valencia-Díaz, Alfonso Leija-Salas, Edgar R. Díaz-García, Pilar Nicasio-Torres, María Del Carmen Gutiérrez-Villafuerte, Jaime Tortoriello-García, Jesús Arellano-García

**Affiliations:** 1 Centro de Investigación en Biotecnología, Universidad Autónoma del Estado de Morelos, Av. Universidad 1001 Col. Chamilpa C.P., Cuernavaca 62209, Morelos, Mexico; norelyvargasmorales@gmail.com (N.V.-M.); iperea@uaem.mx (I.P.-A.); susana.valencia@uaem.mx (S.V.-D.); carmengu@uaem.mx (M.D.C.G.-V.); 2 Centro de Desarrollo de Productos Bióticos, Instituto Politécnico Nacional, Carretera Yautepec-Jojutla Km 6, Calle CEPROBI No. 8, Colonia San Isidro C.P., Yautepec 62731, Morelos, Mexico; 3 Facultad de Medicina, Universidad Autónoma del Estado de Morelos, Iztatzíhuatl esq. Leñeros s/n Col. Los Volcanes C.P., Cuernavaca 62350, Morelos, Mexico; janeth@colpos.mx; 4 Centro de Ciencias Genómicas, Universidad Nacional Autónoma de México, Av. Universidad s/n Col. Chamilpa C.P., Cuernavaca 62210, Morelos, Mexico; leija@ccg.unam.mx; 5 Centro de Investigación Biomédica del Sur, Instituto Mexicano del Seguro Social, Argentina No. 1 Col. Centro C.P., Xochitepec 62790, Morelos, Mexico; edgarrdg@hotmail.com (E.R.D.-G.); pilar_nicasio@hotmail.com (P.N.-T.); jtortora2@yahoo.es (J.T.-G.)

**Keywords:** organogenesis, somatic embryogenesis, *Lopezia racemosa*, genetic transformation, cancer cell lines

## Abstract

A histological analysis was performed with the aim of elucidating the spontaneous regeneration process of the hairy root lines LRT 2.3 and LRT 6.4, derived from *Lopezia racemosa* leaf explants and genetically transformed with the *Agrobacterium rhizogenes* strain ATCC15834/pTDT. The analysis showed both lines regenerate via indirect somatic embryogenesis; LRT 6.4 also regenerated by direct organogenesis. The morphogenic characteristics of the regenerated plantlets from both lines showed the typical characteristics, described previously, including a higher number of axillary shoot formation, short internodes, and plagiotropic roots compared with wild-type seedlings. The regeneration process occurred without the addition of plant growth regulators and was linked to the sucrose concentration in the culture medium. Reducing the sucrose concentration from 3% to 2%, 1%, and 0.5% increased the regeneration rate in LRT 6.4; the effect was less pronounced in LRT 2.3. The cytotoxic activity of different organic extracts obtained from roots and shoots were evaluated in the cancer cell lines HeLa (cervical carcinoma), HCT-15 (colon adenocarcinoma), and OVCAR (ovary carcinoma). The hexane and dichloromethane extracts from roots of both lines showed cytotoxic activity against the HeLa cell line. Only the dichloromethane extract from the roots of PLRT 2.3 showed cytotoxic activity against the OVCAR cell line. None of the methanol extracts showed cytotoxic activity, nor the shoot extracts from any solvent.

## 1. Introduction

*Lopezia racemosa* Cav. is a plant species used in traditional Mexican medicine to cope with a number of illnesses related to the inflammatory process and cancer. It is actually commonly known as the ‘cancer herb’ or ‘pouch herb’. Phytochemical studies from wild plants and in vitro cultures have shown that this species possesses two phytosterols, LR1 and LR2, with anti-inflammatory and cytotoxic activities [1,2]. Moreno-Anzúrez et al. [3] reported on the genetic transformation of this plant for which several hairy root lines were obtained by infecting leaf explants with the *Agrobacterium rhizogenes* ATCC15834/pTDT strain. They also reported the isolation of a new phytosterol named LR3, which possesses higher anti-inflammatory and cytotoxic activities than LR1 and LR2. The hairy root lines LRT 2.3 and LRT 6.4 spontaneously regenerated plantlets after one year of continuous sub-culturing. It is known that in in vitro cultures, the nature and concentration of the carbon source in the culture medium have an effect on growth and morphogenesis, not only due to its energetic value, but also due to varying osmotic potential [4]. The responsiveness of each plant species is also a factor, as well as the culture conditions, the explant, and the *A. rhizogenes* strain used [5].

Obtaining hairy roots by genetic transformation with *A. rhizogenes* is the result of the transfer from the bacteria of a fragment of specific DNA, the T-DNA (Transferred DNA) that is a part of the Ri (Root induction) plasmid [6], and integration into the plant genome. The use of hairy root cultures for the production of secondary metabolites of pharmacological importance has several advantages: hairy roots (1) can grow without the addition of plant growth regulators (PGRs) to the culture medium, (2) show genetic and metabolic stability over long time periods, (3) have a high growth rate and biomass production, and (4) have an enhanced accumulation of the active metabolites as compared with untransformed tissues [7,8]. Some plant species such as *Hypericum perforatum* L. [9], *Tylophora indica* (Burm.f.) Merrill [10], *Plumbago indica* L. [11], and *Bacopa monnieri* (L.) Wettst. [12], among others, have been observed to spontaneously (without adding PGRs) regenerate plantlets from hairy roots.

The T-DNA insertion provokes major changes in an infected plant cell, which grows as a hairy root, and modifies the expression pattern of biosynthetic pathway genes, the secondary metabolite production and, eventually, the pattern of plantlet regeneration from these roots [13]. Morphologically, the regenerated plantlets derived from hairy roots show wrinkled and shortened leaves, reduced apical dominance, shortened internodes, more branching, and a higher number of lateral roots [14,15,16,17].

In the present work, we report on the effect of sucrose concentration on the plantlet regeneration process in the two hairy root lines LRT 2.3 and LRT 6.4, on the histologic analysis performed to elucidate the morphogenetic process of plantlet regeneration in the two lines and, finally, on the cytotoxic activity of two organic extracts obtained from the roots of the regenerated plantlets. None of the methanol extracts from roots and shoots showed cytotoxic activity, nor the shoot extracts from any organic solvent.

## 2. Results and Discussion

### 2.1. Effect of Sucrose Concentration

The regeneration responses of the two hairy root lines to the sucrose concentrations differed (Table 1 and Figures S1 and S2). The hairy roots of the LRT 2.3 line cultivated in MS/B5 media supplemented with 1.0% and 0.5% sucrose did not show a morphogenetic response; instead, they showed apparent accumulation of phenolics and did not grow, whereas the hairy roots from the LRT 6.4 line had a morphogenetic response at the same sucrose concentrations. However, when the hairy roots of LRT 2.3 were cultured in 3% and 2% sucrose, we observed callus formation and somatic embryos at different developmental stages (globular, heart, and torpedo) on the surface of the callus. The subsequent development of embryos or plantlet formation was observed at 20 days post-planting (dpp) in 3% sucrose MS/B5 medium and at 25 dpp in 2% sucrose MS/B5 medium (Figure S1).

In the case of the hairy root line LRT 6.4, it showed spontaneous regeneration at all sucrose concentrations tested. First, we observed callus formation and the subsequent spontaneous development of white and translucent somatic embryos at different developmental stages (globular, heart, and torpedo) on the surface callus (Supplementary Figure S2). The further development of shoots and plantlets was observed at 15 dpp in 0.5% and 1% sucrose MS/B5 medium and in 2% and 3% sucrose MS/B5 medium was observed at 20 dpp.

Comparing the hairy root lines, LRT 6.4 showed a higher rate of regeneration (Table 1). While reducing the sucrose concentration to 2% in the LRT 2.3 line increased the rate of regeneration, further reduction inhibited the regeneration process. In contrast, the LRT 6.4 line showed higher regeneration rates at sucrose concentrations lower than 2%, with the best results obtained at the 1% and 0.5% concentrations. Vinterhalter et al. [9] reported similar results from the hairy roots of *H. perforatum*, with higher regeneration rates when cultivated in MS medium with 1% and 2% sucrose and inhibited regeneration at higher sucrose concentrations. The differential response between the two hairy root lines could be related to the site of T-DNA insertion, the number of T-DNA insertions, or the expression level of some of the *rol* genes inserted, as suggested by Shkryl et al. [18]. Altamura [19] reported that *rol B* gene expression induces the production of new meristems followed by organogenesis and suggested that the depletion of nutrients in the culture medium and an imbalance of PGRs in the transformed tissues could be related to the spontaneous regeneration of plantlets. An analysis of the *rol* gene expression levels in the hairy root lines (LRT 2.3 and LRT 6.4) cultivated under the different sucrose concentrations in MS/B5 medium might contribute to clarifying their different responses.

### 2.2. Histological Analysis

**Figure 1:**
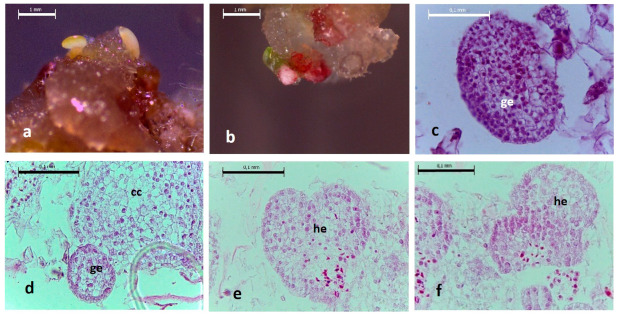
Histological images of embryogenic callus derived from *L. racemosa* hairy roots of the line LRT 2.3. (**a**) embryogenic callus at 10 dpp on MS/B5 medium supplemented with 2.0% sucrose; (**b**) embryogenic callus at 15 dpp on MS/B5 medium supplemented with 3.0% sucrose; (**c**) somatic embryo at globular stage (ge) 10 dpp; (**d**) Compact callus (cc) and somatic embryo at globular stage (ge) 10 dpp; (**e**,**f**) somatic embryos at heart step (he) 15 dpp. (**a**,**b**) (20×) scale bar 1.0 mm, (**c**–**f**) (40×) scale bar 0.1 mm.

The histological analysis of the callus samples derived from the hairy root line LRT 2.3 (Figure 1a,b) showed the presence of a compact callus (Figure 1d) containing cells of different sizes and the development of somatic embryos at the globular and heart stages (Figure 1c–f). Meristematic cells and organized tissues were also observed.

**Figure 2:**
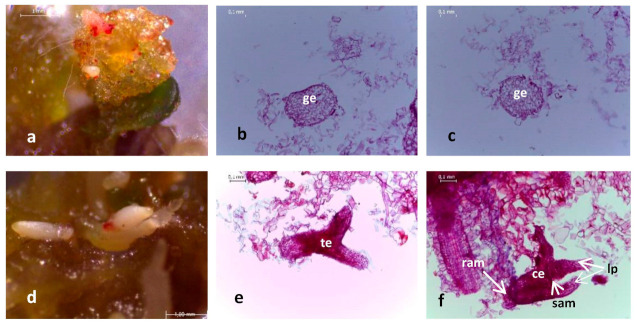
Histological images of embryogenic callus derived from the hairy root line LRT 6.4 of *L. racemosa.* (**a**) embryogenic callus at 5 dpp on MS/B5 medium with 1.0% sucrose; (**b**,**c**) somatic embryos at globular stage (ge); (**d**) embryogenic callus at 15 dpp on MS/B5 medium with 3.0% sucrose; (**e**) somatic embryo at torpedo stage (te); (**f**) somatic embryo at cotyledon stage (ce) showing both, shoot apical meristem (sam) and root apical meristem (ram), leaf primordia (lp) are present as well. (**a**) (35×) and (**d**) (20×) scale bar 1.0 mm, (**b**,**c**) and (**e**,**f**) (10×) scale bar 0.1 mm.

**Figure 3:**
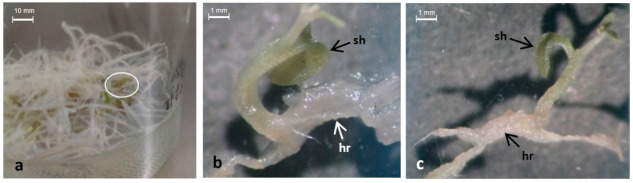
Stereoscopic images of shoots directly regenerated from hairy roots of the line LRT 6.4 of *L. racemosa.* (**a**) Oval shows regenerating hairy roots (hr) cultured on MS/B5 medium with 3.0% sucrose; (**b**,**c**) directly regenerated shoots (sh) showing the connection with its parent tissue (hr) 4× scale bar 10 mm (**a**) and 1 mm (**b**,**c**).

Regarding the line LRT 6.4, the histological analysis of the hairy root-derived callus samples (Figure 2a,d) showed the development of somatic embryos at the globular (Figure 2b,c), torpedo (Figure 2e), and cotyledon (Figure 2f) stages. The last stage showed the shoot apical meristem, the root apical meristem, and leaf primordia. This hairy root line also displayed shoot regeneration without previous callus formation. The direct emergence of shoots from the parent hairy root tissue, which was observed by a stereoscopic microscope, implies a direct vascular connection between them (Figure 3).

With these results, we can assess that the regeneration process in both hairy root lines (LRT 2.3 and LRT 6.4) occur via indirect somatic embryogenesis, as there was callus formation before the appearance of embryos; however, in the case of LRT 6.4, the regeneration process also occurred via direct organogenesis. Plantlet regeneration from hairy root cultures via somatic embryogenesis has previously been reported for several plant species, including *Cucurbita pepo* L. [20], Panax ginseng C. A. Mey. [21], *Beta vulgaris* L. [22], Gentiana macrophylla Pall. [23], and Salvia miltiorrhiza Bunge [17], among others. Such regeneration processes were achieved by the addition of PGRs, but to the best of our knowledge, spontaneous regeneration via somatic embryogenesis without the addition of PGRs has not been reported previously. However, spontaneous plantlet regeneration directly from hairy root cultures has been reported several times, and may be dependent on the photoperiod. *Lotus corniculatus* L. [24], Ophiorrhiza pumila Champ. Ex Benth. [25], *Plumbago indica* L. [11], and *Rauvolfia serpentina* (L.) Benth. ex. Kurz [26] regenerated via direct organogenesis when the hairy roots were incubated under continuous light conditions, while Centaurium erythraea Rafn [27] plantlets regenerated via direct organogenesis when the hairy root cultures were incubated under a 16/8 h light/dark photoperiod. Other plant species that have spontaneously regenerated via direct organogenesis are *H. perforatum* [9], *T. indica* [10], and *B. monnieri* [12].

### 2.3. PCR Analysis

**Figure 4:**
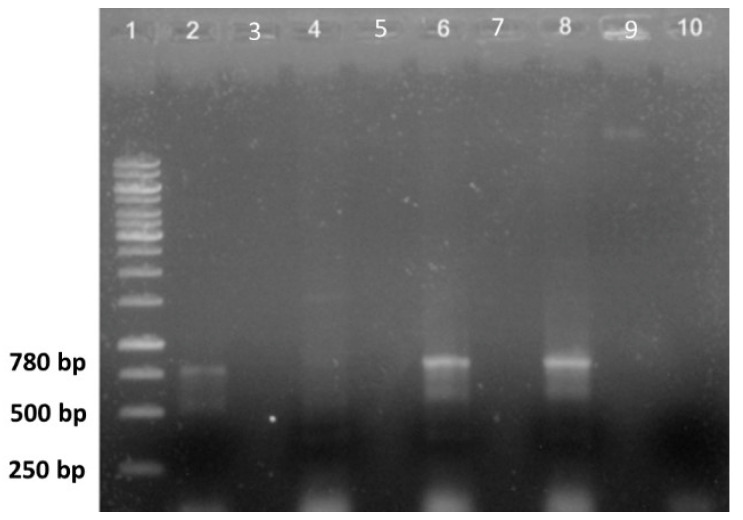
PCR amplification products of the *rol B* gen: lane (1) 1 Kb marker; lane (2) total DNA from *A. rhizogenes* ATCC 15834/pTDT strain; lane (3) empty; lane (4) total DNA from a wild type seedling *L. racemosa*; lane (5) empty; lane (6) total DNA of regenerated plantlet LRT 2.3; lane (7) empty; lane (8) total DNA of regenerated plantlet LRT 6.4; lane (9) empty and lane (10) negative control (water).

The PCR analysis of total DNA samples extracted from the shoots of regenerated plantlets from both hairy root lines (PLRT 2.3 and PLRT 6.4) confirmed the presence of a DNA fragment of 780 base pairs (bp), which corresponds to the PCR product of the rol B gene (Figure 4). As expected, there was no PCR amplification with the negative control (no DNA template) or the genomic DNA extracted from the untransformed seedlings of *L. racemosa.* The positive control, total DNA extracted from *A. rhizogenes* strain ATCC15834/pTDT, also amplified the 780 bp fragment.

### 2.4. Statistical Analysis of the Morphological Characteristics

**Figure 5:**
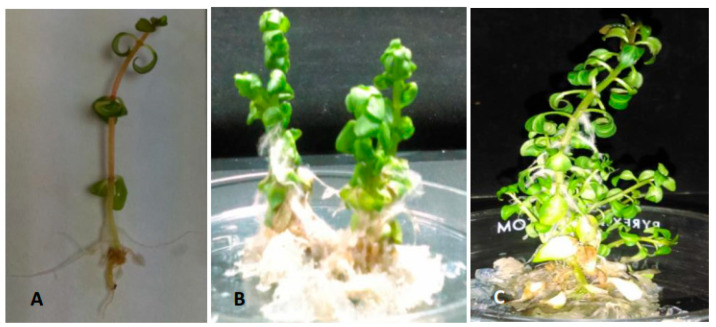
Morphologic characteristics of WT seedlings and regenerated plantlets derived from the transformed hairy root lines of *L. racemosa*. (**A**) WT seedling; (**B**) plantlet derived from the line LRT 2.3; (**C**) plantlet derived from the line LRT 6.4.

The plantlets regenerated from the hairy root lines LRT 2.3 and LRT 6.4 showed the morphological characteristics typical of ‘hairy root syndrome’, with short internodes, a greater number of leaves, and plagiotropic adventitious roots (Figure 5). Such characteristics, which are not present in wild-type (WT) seedlings and plants, have been previously reported in plantlets regenerated from hairy roots derived from several plant species. These characteristics have been associated with the expression of the *rol A*, *B*, *C*, and *D* genes present in the T-DNA of *A. rhizogenes*, which are transferred from the bacterium to the plant genome [16,28,29]. It has also been reported that such phenotypic alterations could be associated with the individual or combined expression of the *rol* genes. Short internodes and wrinkled leaves are associated with the *rol A* gene, while the *rol B* gene is linked to smaller stigma and stamens, and *rol C* is related to reduced apical dominance [14,30].

The plantlets regenerated from the two transformed hairy root lines and from WT seedlings showed significant differences in the number of axillary shoots, the number of nodes, and the number of leaves. However, there were no significant differences between the PLRT 2.3 and PLRT 6.4 regenerated plantlets in terms of internode length and leaf length and width, with both plantlets differing in comparison with the untransformed WT seedlings (Table 2).

The presence of wrinkled leaves was not observed in any plantlet derived from the hairy root lines. However, the presence of plagiotropic adventitious roots was observed in both PLRT 2.3 and PLRT 6.4, but absent in the untransformed WT seedlings (Table 2). Similar results were reported by Chaudhuri et al. [10] in *T. indica*, while Majumdar et al. [12] reported the presence of wrinkled, rounded, and smaller leaves compared with WT plants in *B. monnieri*.

### 2.5. Cytotoxic Activity

The results of the cytotoxic activity assays are shown in Table 3. The hexane and dichloromethane extracts from the roots of both PLRT 2.3 and PLRT 6.4 regenerated plantlets showed cytotoxic activity against the HeLa cancer cell line, whereas against the OVCAR cancer cell line, only the dichloromethane extract of the roots of the PLRT 2.3 regenerated plantlet line showed cytotoxic activity. None of the root extracts from either regenerated plantlet line were active against the HCT-15 cancer cell line.

Our results differ from those reported by Moreno-Anzúrez et al. [3], who found that dichloromethane-methanol extracts obtained from the LRT 7.31 and LRT 17.6 hairy root lines showed no cytotoxic activity against the HeLa cancer cell line. The LRT 7.31 hairy root line showed cytotoxic activity against the HCT-15 colon cancer cell line, and the hairy root line LRT 17.6 showed cytotoxic activity against the OVCAR cancer cell line. These differences could first be explained by the idea that each hairy root line is the result of different genetic transformation events altering plant metabolism in different ways. Another factor could be that the solvents used for the extractions also differ. Finally, none of the organic extracts obtained from the shoots of the PLRT 2.3 and PLRT 6.4 lines showed cytotoxic activity. In contrast to this result, Salinas et al. [2] found compounds with anti-inflammatory and cytotoxic activities in the dichloromethane extracts of the shoots from wild plants and in vitro germinated seedlings (data not shown).

## 3. Materials and Methods

### 3.1. Plant Material

The *L. racemose* hairy root lines LRT 2.3 and LRT 6.4 used in this study were previously generated by Moreno-Anzúrez et al. [3], who started the in vitro plantlet cultures from seeds collected by Salinas et al. [2] from 30 km of the Mexico-Cuernavaca federal road in Morelos, Mexico. These hairy root lines have been sub-cultured in vitro for more than 8 years.

### 3.2. Media and Culture Conditions

The plants and roots were cultured and kept in glass bottles with 50 mL of medium semi-solid as described by Moreno-Anzúrez et al. [3]. The culture medium was prepared with the macro- and micro-nutrients of MS [31] medium and the vitamins in Gamborg B5 [32] medium (MS/B5 medium). It was supplemented with 100 mg/L myo-inositol; 30, 20, 10 or 5 g/L sucrose; and 3 g/L Gelzan™ CM (Sigma-Aldrich) as a gelling agent. The same MS/B5 medium without sucrose was used for seed germination. The pH was adjusted to 5.7 ± 0.1 with potassium hydroxide, and the medium was autoclaved at 108 kPa and 121 °C for 20 min. All the cultures were incubated at a constant temperature (25 ± 2 °C) under a 16/8 h light/dark photoperiod using a 27 µmol·m^2^·s^−1^ white light illumination intensity. Each subculture lasted for 30 days. The data obtained were analysed by Chi square test using VassarStats program (VassarStats: Statistical Computation Website).

### 3.3. Histologic Analysis of the Regeneration Process

Hairy roots (4.0 g) from each line were inoculated in Petri dishes containing 25 mL of semisolid MS/B5 medium supplemented with four alternative sucrose concentrations: 3.0%, 2.0%, 1.0%, or 0.5%. Each treatment had three replicates for a total of 12 Petri dishes per hairy root line. The Petri dishes were incubated in a growth chamber under the conditions described in Section 3.2 for 30 days. Every 5 days, each Petri dish was observed under a stereoscopic microscope to register the morphological changes and take samples of the different morphogenetic structures generated in each Petri dish. The samples were fixed in FAA solution (3.6% formaldehyde, 5% glacial acetic acid, and 50% ethanol) to preserve the plant structures for the analysis and histologic characterization.

For the histologic analysis, the collected and fixed samples were dehydrated in an automatic tissue processor (Leica TP1020 Histokinettel Leica Microsystems, Nussloch, Germany) with an increasing ethanol gradient of 70%, 80%, 96%, and 100% ethanol. The tissue was clarified with xylol-ethanol (1:1) and 100% xylene, making two changes at 30 min intervals. The tissues were later embedded in molten paraffin at 60 °C in 1 cm^3^ molds in a paraffin embedding station (Leica EG1140H) and then cooled to produce solidified blocks. Finally, a Leica rotatory microtome (Minot type) was used to cut the paraffin blocks into 10 µm thick sections, which were put onto microscope slides and stained with safranin and fast green for examination under an optical microscope.

### 3.4. DNA Isolation and PCR Analysis

Total DNA was extracted from the shoots of the regenerated plantlets of both hairy root lines (LRT 2.3 and LRT 6.4) and from untransformed plantlets derived from the in vitro germinated seeds of wild plants (WT seedlings) using the cetyl-trimethyl-ammonium bromide (CTAB) method. Briefly, 120 mg of plant material was ground in liquid nitrogen with a mortar and pestle before adding 1.0 mL of STE buffer (0.25 M Sucrose, 0.03 M Tris-HCl and 0.05 M EDTA) and homogenizing the plant material. The homogenized tissue was transferred to 1.5 mL Eppendorf tubes and centrifuged at 2000× *g* for 10 min, discarding the supernatant at the end (repeated twice); 600 μL of 2× CTAB buffer (100 mM Tris-HCl, 1.4 M NaCl, 20 mM EDTA, 2% CTAB, and 2% PVP), 5 μL of 10 mg/mL RNase A, and 1.8 μL of 0.3% β-mercaptoethanol were added and the tubes heated at 60 °C for 20 min; 600 μL of chloroform:octanol (24:1) was then added, and the tubes were centrifuged at 10,000 rpm for 5 min. After recovering the aqueous phase, the DNA was precipitated with ice-cold isopropanol and pelleted by centrifugation at 13,000 rpm for 4 min; the isopropanol was discarded, and the pellet washed twice with 500 μL of 70% ethanol. Finally, the pellet was resuspended in 50 μL of TE buffer (10 mM Tris-HCl, 0.5 mM EDTA, pH 8.0).

The DNA samples were used as template for the PCR analysis to amplify a fragment (780 bp) of the *rolB* gene using the primers 5′-ATGGATCCCAAATTGCTATTCCCCCACGA-3′ and 3′-TTAGGCTTCTTTCATTCGGTTTACTGCAGC-5′, as described by [33]. This gene is transferred to the plant cell during the transformation process and is important in the development of the hairy roots. The PCR amplifications were prepared with a Vivantis kit in 500 µL PCR tubes on ice, with a total reaction volume of 50 µL containing 1 µL DNA template, 5 µL of 10× Taq DNA polymerase reaction buffer, 2 µL of 50 mM MgCl_2_, 1 µL of each primer (10 μM), 2 µL of dNTP mixture (2 mM), 0.5 µL of recombinant Taq DNA polymerase (5U/μL), and 37.5 µL of nuclease-free water. The PCR conditions were one 5 min denaturing cycle at 95 °C; 35 cycles of 1 min at 95 °C, 1 min at 50 °C, and 1 min at 72 °C; and a final extension at 72 °C for 5 min. The PCR products were electrophoresed on 1% agarose gels at 100 V for 60 min and visualized on a UV trans-illuminator (BioDoc-It™ Imaging System, Upland, CA, USA) after staining with ethidium bromide. The GeneRuler™ (Thermo Scientific, Vilnius, Lithuania) 1 kb marker was used as a size reference.

### 3.5. Morphological Characterization of Regenerated Plantlets

The seeds from *L. racemosa* wild plants used in this study were previously collected and reported on by Salinas et al. [2], as were the methods for their surface sterilization and germination. The aim of obtaining aseptic WT seedlings was to have a reference for the morphological comparison of the regenerated plantlets derived from the LRT 2.3 and LRT 6.4 hairy root lines, which were named PLRT 2.3 and PLRT 6.4, respectively. From the germinated seedlings, 14 WT shoots (1.5–2.0 cm long) were chosen, as were 14 axillary shoots (1.5–2.5 cm long) derived from PLRT 2.3 and 14 axillary shoots (3.0–4.0 cm long) derived from PLRT 6.4. Each group had three replicates. All the seedlings and shoots of the regenerated plantlets were sub-cultured into 250 mL flasks containing 50 mL of MS/B5 medium, planting two seedlings or plantlets per flask, and incubated at the same conditions mentioned in Section 3.2. After 30 days, the following morphologic characteristics were recorded: length of the main shoot, number of axillary shoots, number of nodes, length of internodes, number of leaves, and the length and width of the leaves (three leaves from the same height of each seedling or plantlet). We also recorded the average number of seedlings and plantlets that showed or lacked wrinkled leaves, adventitious roots, and plagiotropic roots. The data obtained from the two lines (PLRT 2.3 and PLRT 6.4) and WT seedlings were subjected to a one-way analysis of variance (ANOVA). In the case of statistical differences (*p* < 0.05), a post hoc Tukey test was performed on the data for each line. All statistical analyses were carried out using VassarStats and GraphPad programs.

### 3.6. Obtaining Organic Extracts

The roots and shoots of the *L. racemosa* regenerated plantlets PLRT 2.3 and PLRT 6.4 were collected and dried at room temperature until the weight was constant. Each dry matter sample was then ground and extracted at increasing polarities with hexane, dichloromethane, and methanol (1:10 *w*/*v*) for 72 h. Finally, the solvent in each sample was evaporated at room temperature and the extracted yield recorded.

### 3.7. Cytotoxic Activity

Cytotoxic activity was evaluated in the human cancer cell lines HeLa (cervical carcinoma), HCT-15 (colon adenocarcinoma), and OVCAR (ovary carcinoma), which were cultured in Eagle’s minimum essential medium (MEM) containing 10% fetal bovine serum and incubated at 37 °C in a 5% CO_2_ atmosphere with 100% relative humidity. When the cells reached the log phase, they were transferred to multi-well plates (50,000 cells/mL), and three replicates of each cancer cell line were treated with the vehicle (DMSO, negative control), vinblastine (0.037, 0.028, and 0.039 μg/mL, respectively; positive controls), or three different concentrations of each organic extract (1, 10, and 100 μg/mL). The multi-well plates were incubated for 72 h under the same conditions, after which, the cell concentration for each treatment was determined by quantifying the protein with the Lowry method. The results are expressed as the extract concentration that inhibited growth by 50%, i.e., the median inhibitory concentration (IC_50_) relative to the negative control [34]. Values were estimated from the treatment semi-log concentrations versus the percentage of viable cells.

## 4. Conclusions

This work showed that a reduction in sucrose concentration in the culture medium increased the rate of spontaneous regeneration in both hairy root lines, with higher rates in the LRT 6.4 line. The regeneration process in both hairy root lines was through indirect somatic embryogenesis; however, the LRT 6.4 line also displayed a direct regeneration process. It is important to note that there were differences in the responsiveness of the two hairy root lines (LRT 2.3 and LRT 6.4), which may be related to the insertion site of the T-DNA, the number of T-DNA insertions, or to the expression levels of the *rol* genes inserted. The organic extracts obtained from the shoots of the PLRT 2.3 and PLRT 6.4 regenerated plantlets did not show any cytotoxic activity, nor the methanol extracts obtained from the roots of both plantlet lines. Further work will be necessary to elucidate the nature and structure of the compounds with cytotoxic activity that were present in the organic extracts derived from roots of both plantlet lines, as well as to perform a molecular analysis of the *rol* genes in the hairy root lines and plantlets.

## Figures and Tables

**Table 1:** Chi-Square (*Xi*^2^) statistical analysis of the total number of regenerated plantlets per hairy root line during 30 days on MS/B5 medium containing different sucrose concentrations. It shows significant difference (*p* < 0.01) between both lines. Degrees of freedom (df) = 3.

Sucrose %	LRT 2.3	LRT 6.4
3.0	2	21
2.0	4	22
1.0	0	43
0.5	0	45
Total	6	131

*Xi*^2^ = 12.57, df = 3, *p* < 0.01.

**Table 2:** Data from the ANOVA analysis of the morphological characteristics of the plantlets regenerated from the hairy root lines as compared with WT seedlings after 30 days on culture.

Morphological Characteristics	WT Seedlings	PLRT 2.3 Plantlets	PLRT 6.4 Plantlets
Main shoot length (cm)	3.34 ± 0.19 ^c^*	8.18 ± 0.23 ^b^*	11.04 ± 0.32 ^a^**
Number of axillary shoots	0 ± 0 ^c^	4.47 ± 0.44 ^b^*	10.71 ± 0.62 ^a^**
Number of nodes	4.35 ± 0.13 ^c^**	9.35 ± 0.35 ^b^*	13.14 ± 0.45 ^a^**
Number of leaves	11.5 ± 0.72 ^c^**	93.14 ± 5.60 ^b^**	137.35 ± 5.69 ^a^**
Length of internodes (cm)	0.75 ± 0.04 ^a^*	0.60 ± 0.01 ^b^	0.63 ± 0.01 ^b^
Leaf length (cm)	0.94 ± 0.03 ^b^*	1.52 ± 0.04 ^a^	1.56 ± 0.03 ^a^
Leaf wide (cm)	0.45 ± 0.01 ^b^*	0.70 ± 0.02 ^a^	0.74 ± 0.01 ^a^
Wrinkle leaves (%)	0	0	0
Aerial plagiotropic roots (%)	0	100	100

Significant difference at * *p* < 0.05; ** *p* < 0.01 by one-way ANOVA and Tukey’s test different letters indicate significant difference *p* < 0.05.

**Table 3:** Cytotoxicity of root organic extracts of plantlets (IC_50_) derived from hairy root lines LRT 2.3 and LRT 6.4 of *L. racemosa*. Positive control: Vinblastine.

Plantlet Line	Cancer Cell Lines			
	Organic Extract	HeLa (μg/mL)	HCT-15 (μg/mL)	OVCAR (μg/mL)
PLRT 2.3	Hexane	16.21	˃100	>100
	Dichloromethane	11.83	34.67	14.79
PLRT 6.4	Hexane	19.95	75.85	˃100
	Dichloromethane	12.88	33.88	23.44
Vinblastine	-	0.037	0.028	0.039

Cancer Cell Lines: Cervical carcinoma (HeLa), Colon adenocarcinoma (HCT-15) and Ovary carcinoma (OVCAR).

## Data Availability

Data is contained within the article and supplementary materials.

## Supplementary Materials

The following supporting information can be downloaded at: https://www.mdpi.com/article/10.3390/plants11020150/s1. Figure S1: Regeneration process of plantlets derived from the hairy root line LRT2.3 of *L. racemosa* cultured on MS/B5 medium with two different sucrose concentrations: 2 % and 3 % without PGRs during 30 dpp. M, callus; N, S and T calli with somatic embryos at globular stage; O, somatic embryo between heart and torpedo stage; P, somatic embryo at torpedo stage; U callus; Q, R, V, W and X shoots. Figure S2: Regeneration process of plantlets derived from the hairy root line LRT6.4 of *L. racemosa* cultured on MS/B5 medium containing different sucrose concentrations: 0.5 %, 1.0 %, 2.0 % and 3.0 % without PGRs during 30 dpp. A and G, calli with somatic embryos at globular stage; H, M and S, calli with somatic embryos at heart stage; B and N somatic embryo at torpedo stage; O, somatic embryo at cotyledonary stage; S and U, somatic embryo at heart and early torpedo stage; C to F, I to L, P to R and V to X, shoots.
