# Supplementary material for: Spontaneous Regeneration of Plantlets Derived from Hairy Root Cultures of Lopezia racemosa and the Cytotoxic Activity of Their Organic Extracts
Source: Plants (Basel). 2022 Jan 6;11(2):150. doi: 10.3390/plants11020150 (PMC8780091; doi:10.3390/plants11020150)
Supplement: Supplementary file 1 [file plants-11-00150-s001.zip › plants-1485403-supplementary.pdf]

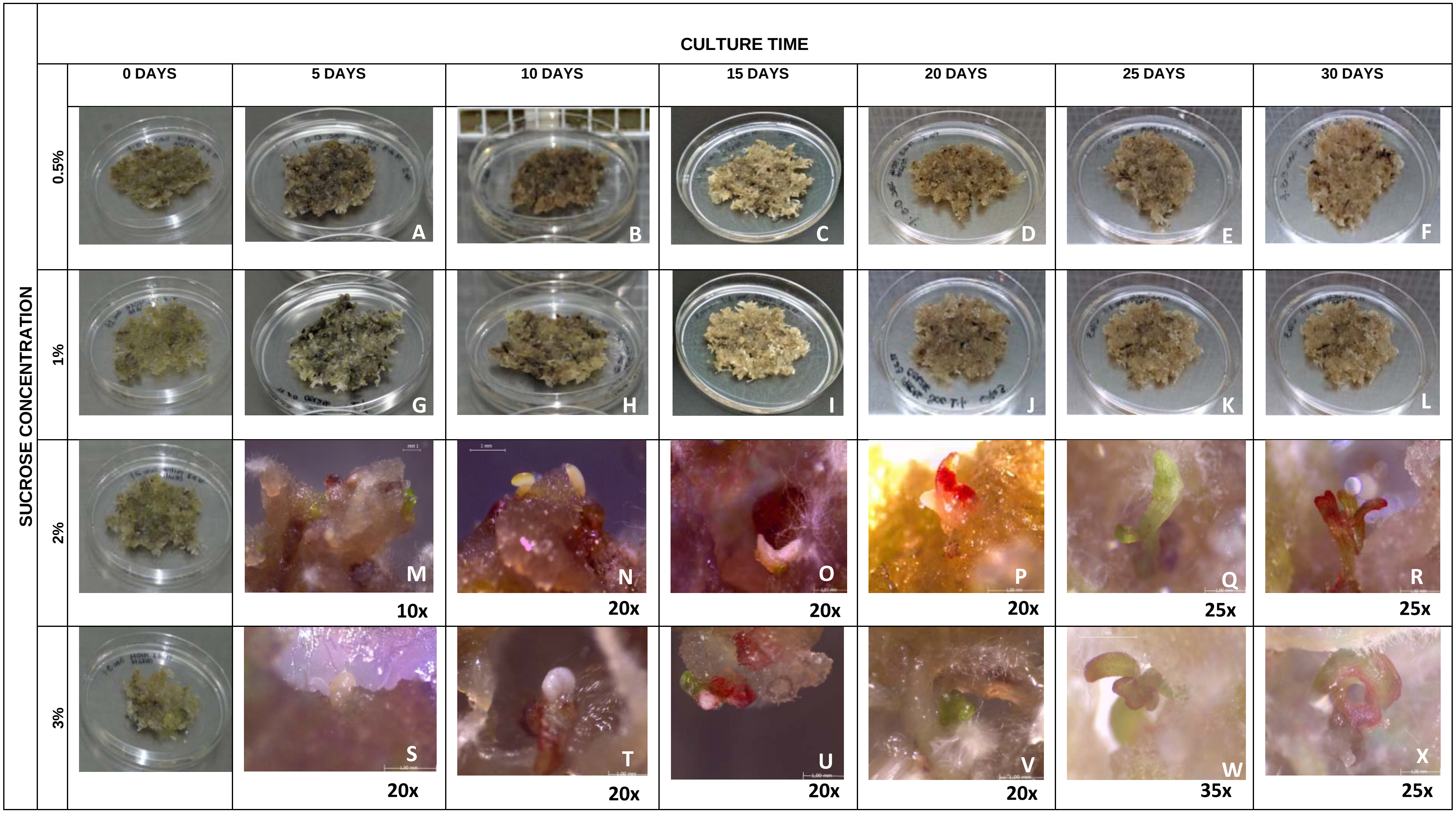

Supplementary figure S1 Regeneration process of plantlets derived from the hairy root line LRT2.3 of *L. racemosa* cultured on MS/B5 medium with two different sucrose concentrations: 2 % and 3 % without PGRs during 30 dpp. **M**, callus; **N**, **S** and **T** calli with somatic embryos at globular stage; **O**, somatic embryo between heart and torpedo stage; **P**, somatic embryo at torpedo stage; **U** callus; **Q**, **R**, **V**, **W** and **X** shoots

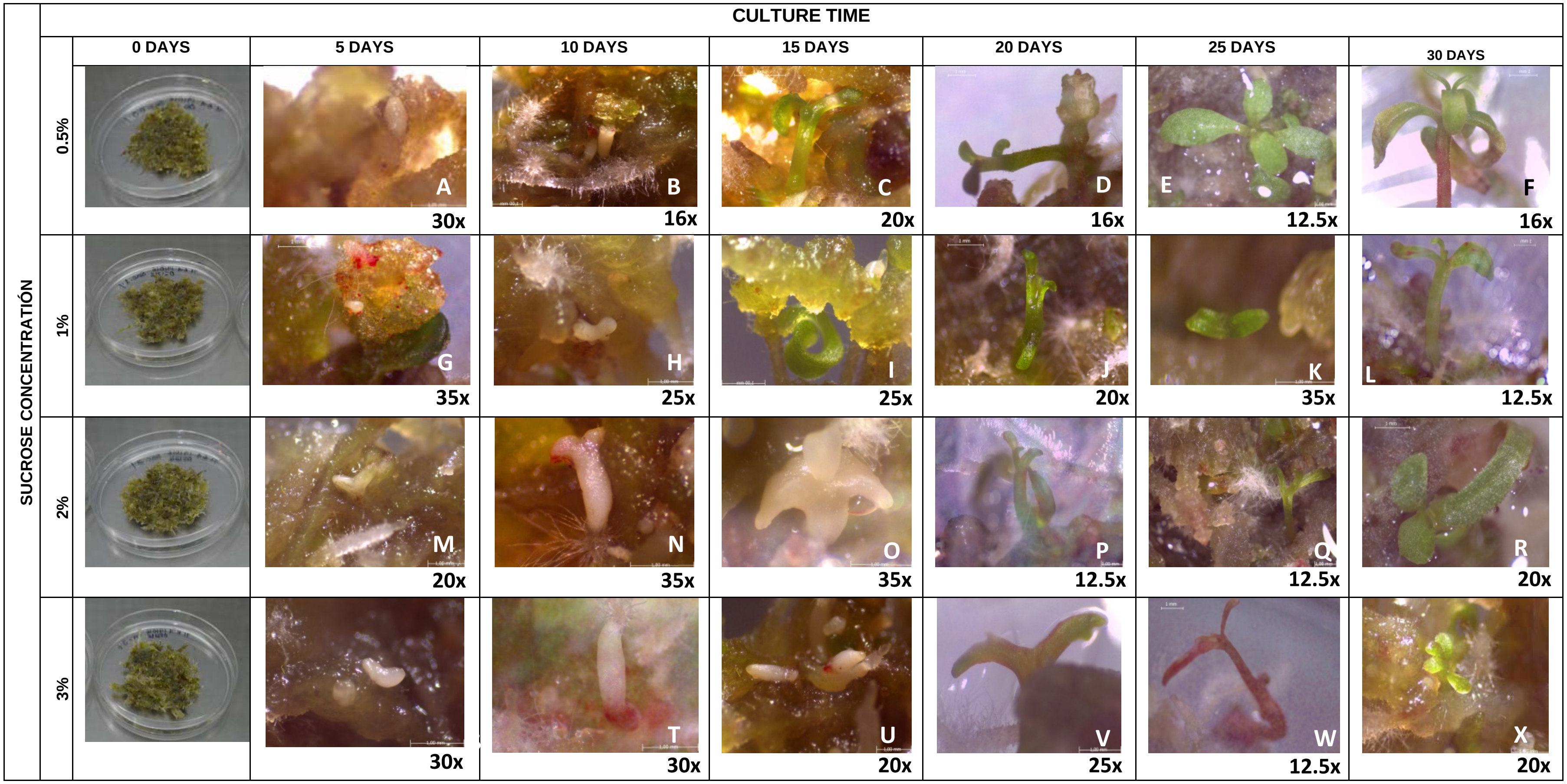

Supplementary figure S2 Regeneration process of plantlets derived from the hairy root line LRT6.4 of *L. racemosa* cultured on MS/B5 medium containing different sucrose concentrations: 0.5 %, 1.0 %, 2.0 % and 3.0 % without PGRs during 30 dpp. **A** and **G,** calli with somatic embryos at globular stage; **H, M** and **S**, calli with somatic embryos at heart stage; **B** and **N** somatic embryo at torpedo stage; **O**, somatic embryo at cotyledonary stage; **S** and **U**, somatic embryo at heart and early torpedo stage; **C** to **F**, **I** to **L**, **P** to **R** and **V** to **X,** shoots
